# Cone Beam Computed Tomography (CBCT)-Based Online Adaptive Radiotherapy for Patients With Metal Implants: An Institutional Experience

**DOI:** 10.7759/cureus.106211

**Published:** 2026-03-31

**Authors:** Fiona Li, Sean Tanny, Wesley Rivais, Olga Dona Lemus, Alexander Podgorsak, Dandan Zheng

**Affiliations:** 1 Radiation Oncology, University of Rochester, Rochester, USA; 2 Radiation Oncology, Radformation, New York, USA; 3 Radiation Oncology, University of Miami, Miami, USA

**Keywords:** ethos-based adaptive radiotherapy, metal artifact, metal implant, online adaptive radiation therapy, workflow

## Abstract

Background

Patients with metal implants (MIs) are often excluded from the current cone beam computed tomography (CBCT)-based online adaptive radiotherapy (oART) workflows because of concerns regarding imaging artifacts, potentially limiting access to this innovative technology.

Purpose

This study aimed to present our institutional experience treating patients with MI using an oART workflow and to evaluate dose accuracy and on-couch treatment time.

Methods

Eight patients treated at the Wilmot Cancer Center (University of Rochester, Rochester, USA) on the Ethos system were retrospectively analyzed, including five with MI and three without MI. During treatment planning, dosimetric avoidance through the MI was implemented to minimize dose deposition within the implants. The necessity of overriding metal artifacts (MAs) surrounding the implants was evaluated using 3D gamma analysis, as well as comparisons of D_max_, D_min_, and D_mean_. The impact of MA on AI-based auto-contouring was assessed by comparing automatically generated contours with expert-modified contours using the Dice similarity coefficient (DSC) and maximum Hausdorff distance (MHD). Contouring times were also compared between patients with and without implants.

Results

Dosimetric avoidance through the MI achieved clinically acceptable plan quality. No clinically meaningful differences in dose distribution were observed between plans with and without MA overrides, suggesting overrides may be unnecessary unless MA overlaps with target volumes. Although the presence of MI and CBCT artifacts affected auto-contouring performance, they did not compromise the feasibility of oART delivery on the Ethos system. Contouring times were comparable between implant and non-implant patients. Overall, DSC and MHD values demonstrated good agreement between auto-contoured and manually edited structures, with the exception of the bladder.

Conclusions

Our proposed workflow demonstrates the feasibility of CBCT-based oART for patients with MIs based on institutional experience. Additional validation may be required to meet institutional standards and policy requirements.

## Introduction

Online adaptive radiotherapy (oART) is one of the most recent advancements in radiation oncology and is characterized by real-time online adaptation of treatment plans based on daily patient anatomy. This is particularly advantageous in regions subject to significant inter-fractional motion, where daily adaptation helps ensure adequate target dose coverage while minimizing dose to nearby organs at risk (OARs).

Ethos (Varian Medical Systems, Palo Alto, CA, USA) is a radiotherapy platform that provides cone beam computed tomography (CBCT)-guided oART capabilities. oART remains an active area of research and development within radiation oncology. Compared with other treatment planning systems (TPS), the Ethos treatment planning management system (TPMS) limits user intervention during treatment planning in favor of automation, with decision-making largely guided by artificial intelligence (AI) [1]. Consequently, the oART workflow relies heavily on AI-based algorithms to compress the entire adaptive process into a short on-couch treatment time. This workflow is more complex than conventional radiotherapy delivery and begins with daily CBCT acquisition, followed by AI-based auto-contouring of influencer structures. These structures, which are closest to the target, have the greatest impact on target contouring and can be reviewed and edited by clinicians. Target contours are subsequently generated based on the deformation of the planning CT to the daily CBCT and may also be manually modified. Together, the target and normal tissue contours comprise the session model for adaptive planning.

Because AI-based contouring relies on daily CBCT images, the presence of high atomic number (high-Z) materials, such as hip prostheses, dental implants, or spinal fixation hardware, can introduce hyperdense and hypodense artifacts that degrade Hounsfield unit (HU) accuracy and compromise dose calculation near the implant [2-4]. These artifacts can also adversely affect auto-segmentation of influencer structures and target generation. As a result, patients with metal implants (MIs) are frequently excluded from oART workflows to mitigate planning uncertainty and avoid complications during the time-constrained on-couch adaptive process.

In conventional non-adaptive planning workflows, HU values within MIs and associated streak artifacts are commonly corrected manually [5-6]. The AAPM Task Group 63 (TG-63) recommends avoiding beam paths through metallic prostheses due to dosimetric uncertainties associated with metallic inhomogeneities [6]. However, with the advent of advanced dose calculation algorithms, such as AcurosXB (AXB), the relevance of TG-63 recommendations with respect to dose accuracy may be diminished. Ethos TPMS employs AXB, a grid-based Boltzmann solver with accuracy comparable to fast Monte Carlo methods, which is also implemented in contemporary commercial TPS platforms. Nevertheless, the adaptive planning workflow in Ethos TPMS is highly streamlined, offering less customization than conventional planning systems.

Plan optimization in Ethos TPMS is performed using the Intelligent Optimization Engine (IOE), which automatically generates treatment plans based on user-defined clinical goals and priorities [7]. Standardized beam geometries consisting of seven, nine, or 12 equally spaced coplanar beams are automatically generated. Customized beam arrangements are not directly supported and require workarounds, such as importing beam geometries from third-party TPS platforms. In addition, sector-based beam avoidance or OAR-based beam blocking is not available. Consequently, modulation of dose through MIs must be achieved indirectly, for example, by imposing strict dose constraints on implant structures to enforce dosimetric avoidance.

In this work, we present an oART workflow derived from our institutional experience treating patients with MIs, including dental, hip, and spinal stabilization hardware. Recommendations for managing patients with MIs in CBCT-based oART are proposed, with consideration of dose accuracy, on-couch treatment time, and overall workflow robustness.

## Materials and methods

Study cohort

A total of eight patients treated at the Wilmot Cancer Center (University of Rochester, Rochester, USA) with the Ethos linear accelerator between 2022 and 2024 were randomly selected for the study (Table 1). Patients were selected based on the presence of a MI in the treatment region. Five patients had some form of MI, three of whom underwent adaptive radiotherapy. The remaining three patients did not have MIs and were included for comparison of the proposed workflow. Non-adaptive patients with implants were randomly selected from the same time period. Only patients receiving adaptive radiotherapy for the prostate bed with nodal involvement were included in the adaptive analysis.

**Table 1:** Patient cohorts with their respective demographics

Patient #	Age	Sex	Treatment site	Metal implant	oART
1	79	M	Prostate bed + nodes	Unilateral Hip Implant	Yes
2	68	M	Prostate bed + nodes	Bilateral Hip Implant	Yes
3	61	M	Prostate bed + nodes	Spine Stabilization	Yes
4	65	M	Prostate bed + nodes	No Implant	Yes
5	68	M	Prostate bed + nodes	No Implant	Yes
6	68	M	Prostate bed + nodes	No Implant	Yes
7	54	F	Nasopharynx	Dental Implant	No
8	67	F	Head and neck	Dental Implant	No

All patients underwent CT simulation using standard reconstruction settings on a GE Lightspeed scanner (GE Healthcare, IL, USA). Patients included in the study were treated using the Ethos V1.0 (Varian Medical Systems, Palo Alto, CA, USA) system. Pelvic CBCT images were acquired using 125 kV with a tube current of 80 mA (760 mAs). For head-and-neck (HN) cases, CBCT acquisition parameters were 125 kV and 35 mA (300 mAs). Due to the unavailability of a metal artifact reduction (MAR) algorithm in the CT scanner, an image-only session using Ethos v2.0 HyperSight was scheduled for contouring the target and OARs for the bilateral hip implant patient.

Treatment planning with hip implants

Among the six adaptive patient cohorts, two patients with hip implants were used for the sector avoidance study. For simplicity, in these multi-phase treatments, all analyses were performed on phase 1 treatment delivery, and the phase 2 prostate bed dose boost was excluded. For phase 1, the prescription doses to the targets (prostate bed and nodal volumes) were 45 Gy in 25 fractions and 48.6 Gy in 28 fractions for the two patients, respectively.

For treatment planning, the contoured hip implant was overridden to titanium material. Plans were optimized on the Ethos TPMS platform (v1.1) using 12 equidistant intensity-modulated radiation therapy (IMRT) fields, as non-equidistant beam arrangements are not directly supported by the Ethos TPMS optimizer.

Because the structure avoidance technique available in routine planning is not implemented in Ethos, alternative strategies were developed to minimize dose through the hip implants. For patients with unilateral hip implants, the implant structure was uniformly expanded by 1 cm, except in the medial and lateral directions, where expansions of 5 and 3 mm were applied, respectively. A low maximum dose constraint was imposed (D0.03cc ≤ 2430 cGy, corresponding to 90 cGy per fraction). For patients with bilateral hip implants, the implant structures were expanded by 3 mm in the anterior-posterior direction, with a dosimetric constraint of mean dose (D_mean_) ≤ 714 cGy (28.6 cGy per fraction).

The effectiveness of the dosimetric avoidance strategies was evaluated by comparing the dose delivered through the implants in adaptive versus scheduled treatment sections. The dosimetric metrics analyzed were D_mean_ and dose maximum (D_max_). Statistical significance between datasets was assessed using Student’s t-test, with a threshold of p < 0.05. All statistical analyses were performed using Microsoft Excel (Microsoft® Corp., Redmond, WA).

Dosimetric impact of artifact overrides

The dosimetric impact of artifact overrides was evaluated in a retrospective analysis. This analysis was performed on the Eclipse treatment planning system, which allows direct modification of the HU values associated with imaging artifacts. In the clinical plans, imaging artifacts arising from the implants were overridden to a tissue density of 0. The impact of artifact override was assessed by comparing plans generated with and without artifact overrides for all patients with implants. Plans with artifact overrides were recalculated after the removal of the HU overrides to generate corresponding plans without artifact correction.

All Eclipse plans were calculated using the AcurosXB dose calculation algorithm (version 15.6.06). Dosimetric comparisons between plans included D_mean_, minimum dose (D_min_), D_max_, and dose-volume histograms (DVHs). Dose distribution maps were compared using SNC Patient software (Sun Nuclear, Melbourne, FL; version 8.5.0) employing a three-dimensional gamma analysis with criteria of 2%/2 mm. Statistical significance was assessed using the Wilcoxon signed-rank test with a significance level of α = 0.05.

Propagation of artifact contours between fractions in oART was visualized by exporting the contours to third-party software, including Velocity (Varian Medical Systems, Palo Alto, CA, USA) and Eclipse. Contour propagation accuracy was evaluated by comparing the reference (planning) contours with the daily on-couch deformed contours. This analysis was performed for patients with bilateral hip implants. The metrics used were the Dice similarity coefficient (DSC) and the maximum Hausdorff distance (MHD), both calculated using Velocity.

Effects of metal artifacts on auto-contouring

The effect of metal artifacts on the accuracy of auto-contouring for influencer structures was evaluated across two adaptive sessions in patients treated for prostate bed and pelvic lymph nodal targets, including one patient with a spine implant and one with a unilateral hip implant. The patient with bilateral hip implants was excluded from the auto-contouring analysis due to severe dark artifacts between the two implants caused by beam hardening.

An emulator was used to reproduce the clinical on-couch adaptive workflow and to generate automatically contoured, unedited influencer, target, and OAR structures. Contours reviewed and manually edited by expert clinicians during actual adaptive sessions, along with the unedited auto-contours generated by the emulator, were imported into Velocity for analysis. The impact of artifacts on auto-contouring performance was assessed by quantifying agreement between the automatically generated contours and the clinically adjusted contours using the DSC and MHD for both target volumes and OARs.

In addition, contouring time during real on-couch adaptive sessions for all phase 1 fractions was compared between patients with and without MIs to evaluate the effect of implant-related artifacts on workflow efficiency. Statistical significance between mean contouring durations was assessed using Student’s t-test.

## Results

**Figure 1:**
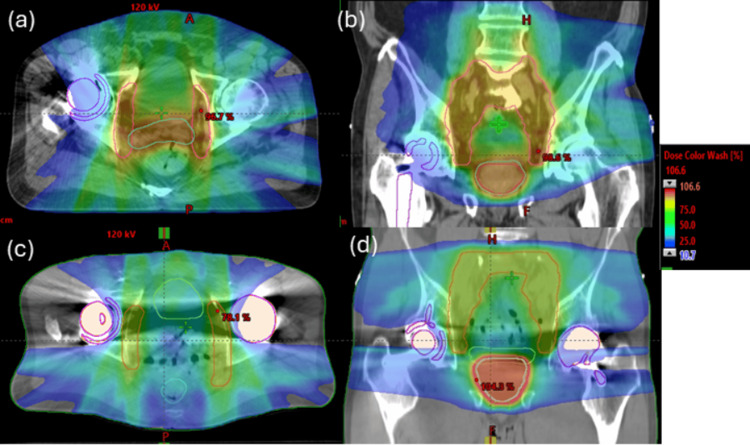
Axial and coronal views of the dose distribution achieved with dosimetric avoidance through the hip implant for two patients with (a, b) unilateral (patient #1) and (c, d) bilateral implants (patient #2) Snapshot images from Eclipse (Siemens Healthineers, Palo Alto, CA)

Figure 1 shows the planned dose distributions for two patients with (Figure 1a, 1b) unilateral and (Figure 1c, 1d) bilateral hip implants treated using dosimetric avoidance. Successful dose avoidance through the implants is demonstrated by the 20% isodose line in the color wash and, for the unilateral implant case, by comparison with the contralateral femur.

**Figure 2:**
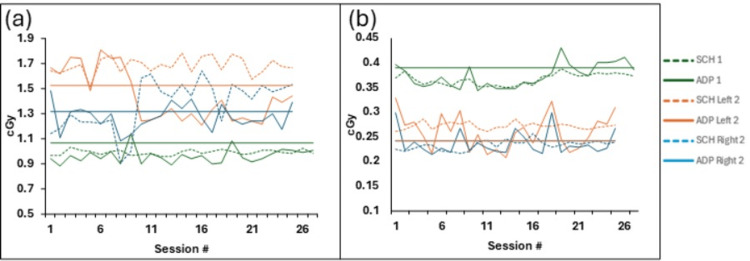
Comparison of (a) Dmax and (b) Dmean in the contoured implants for each scheduled (SCH) and adaptive (ADP) sessions for two patients, as indicated by 1 and 2. Patient 1 had a unilateral hip implant, and Patient 2 had a bilateral hip implant. The solid straight line indicates the reference dose. Image created by the authors with MS Excel (Microsoft Corp., WA, USA)

Figures 2a-2b show the D_max_ and D_mean_ values of the implant contours for the scheduled and adaptive plans in the two hip implant patients. Table 2 shows the mean of the differences between the scheduled and the adaptive plan and its standard deviation. No significant difference was observed in the mean D_mean_ between the scheduled and adapted sessions, whereas a statistically significant difference was found for D_max_.

**Table 2:** Mean absolute difference in the contoured implants between the adaptive and scheduled plan for Dmean (Gy) and Dmax (Gy). Statistical analysis were performed using student's t-test.

Patient #	Metrics	Mean ± STD	p-value	t statistics
1	D_mean_	0.012 ± 0.011	0.15	-1.47
	D_max_	0.054 ± 0.041	0.02	2.39
2 (Left)	D_mean_	0.032 ± 0.020	0.1	1.68
	D_max_	0.275 ± 0.182	<0.05	5.74
2 (Right)	D_mean_	0.020 ± 0.020	0.51	-0.67
	D_max_	0.187 ± 0.123	0.02	2.36

Metal artifact override versus no override

**Figure 3:**
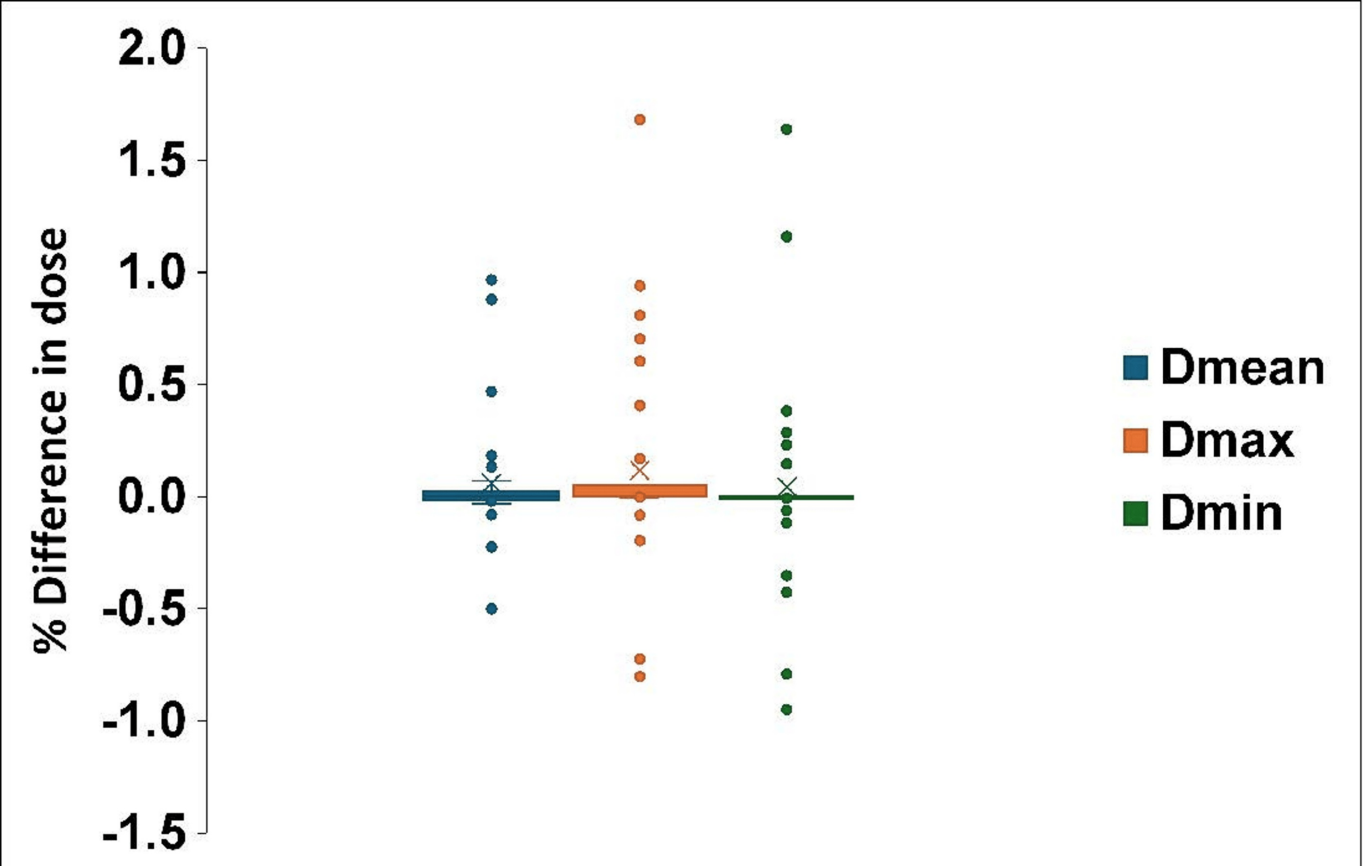
Calculated dose differences with and without artifact override. The plot shows the percentage differences in doses with and without Hounsfield unit (HU) overrides for the contoured artifacts. Each point represents the individual plan for five patients (#1-3, 7, and 8) averaged over the planning target volumes (PTVs) and the organs at risk (OARs). Image created by the authors with MS Excel (Microsoft Corp., WA, USA)

Overriding the artifacts surrounding the MIs did not result in significant dosimetric differences in the target volumes or surrounding tissues (Figure 3). The D_mean_, D_max_, and D_min_ did not differ significantly, as assessed using the Wilcoxon signed-rank test. The DVHs for patients without artifacts overlapping the targets were similar.

**Figure 4:**
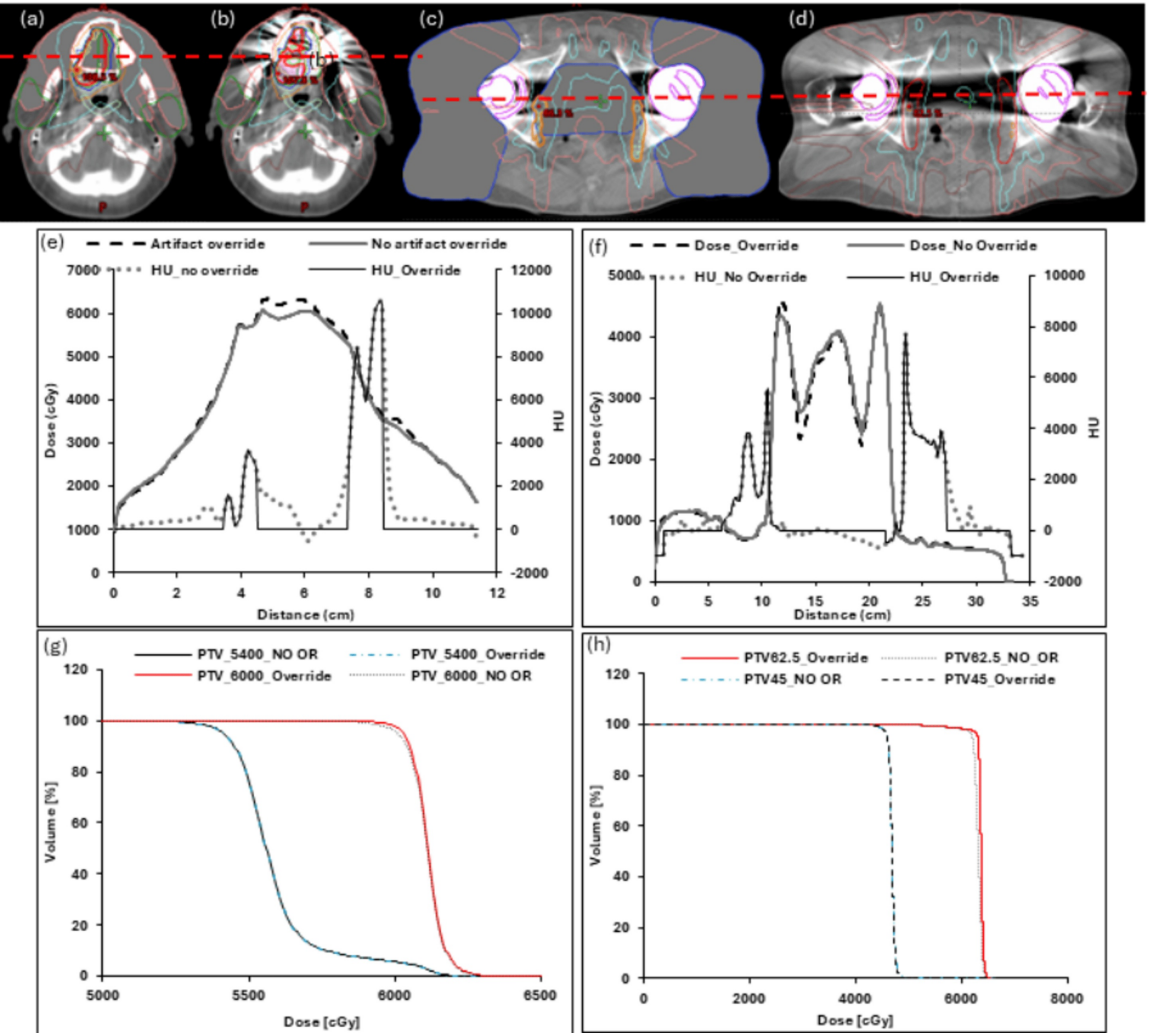
Dosimetric differences for cases where the artifact overlaps with the regions of interest. (a) and (b): Regions where the artifact overlaps with the target in the oral cavity (patient #8), with artifacts (a) overridden and (b) not overridden. (c) and (d): Target overlap in the bilateral hip implant patient #2 with artifacts (c) overridden and (d) not overridden. (e) and (f): Dose profiles corresponding to the red lines through each set of images (a-d) along with the differences in Hounsfield unit (HU) across the profiles. The corresponding differences in DVH due to artifact overrides for the targets are shown in (g) and (h) for the oral cavity and hip implants, respectively. (a)-(d) are snapshot images from Eclipse (Siemens Healthineers, Palo Alto, CA). The graphs are created by the authors with MS Excel (Microsoft Corp., WA, USA).

For most patients, the PTV did not overlap with artifact regions; exceptions included the nasopharyngeal and bilateral hip implant cases (Figure 4). In the nasopharyngeal case, the DVH showed 1-2% differences in V100% and V99% due to artifact overriding. By contrast, the bilateral hip implant case exhibited larger dose differences, with a 12% difference in V100% and a 2% difference in V99%. Figures 4a-4d illustrate the regions where the PTV overlaps with metal artifacts for the oral cavity and bilateral hip implant cases. As expected, the largest dose discrepancies occurred in regions with the greatest HU differences.

Gamma analysis using 2%/2 mm criteria yielded volumetric passing rates greater than 95% for both cases. The DSC and mean MHD for artifact contour propagation between the planning CT and daily on-couch sessions were 0.82 ± 0.01 and 20.61 ± 3.76 mm, respectively.

Effect of metal artifacts on auto-contouring

**Figure 5:**
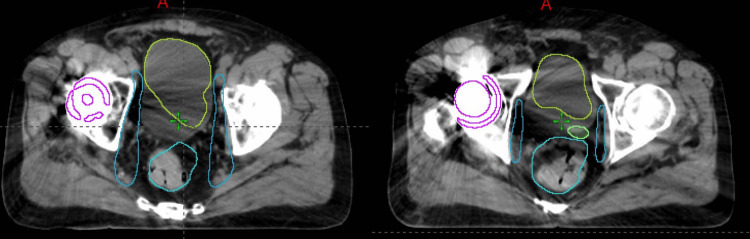
Effects of metal artifacts on on-couch influencer auto-contouring of the bladder (green), rectum (blue), and seminal vesicle (yellow) on two different axial slices of the same patient (patient #1). Magenta is the auto-contoured hip implant. Snapshot images from Eclipse (Siemens Healthineers, Palo Alto, CA)

**Figure 6:**
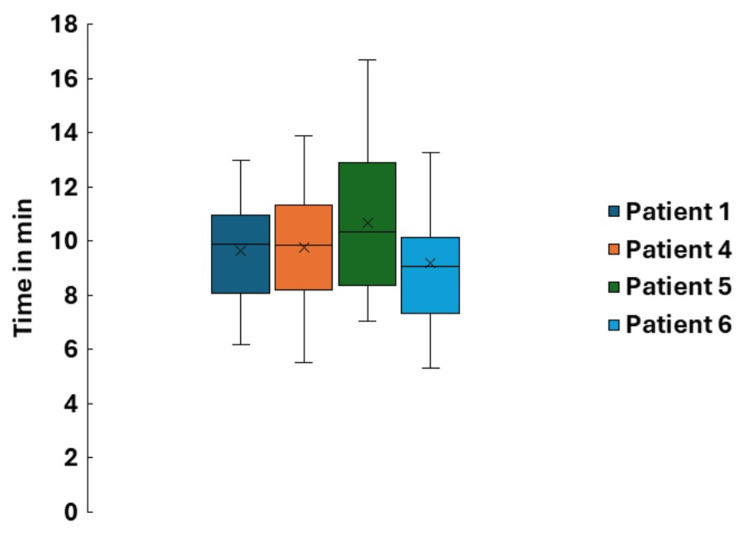
Plot of the distribution of contouring time in each session for the four analyzed patients. All four patients received conventional-fractionation adaptive radiotherapy (ART) to the prostate bed and lymph nodes. Patient 1 had a hip implant, and the other three patients had no implants. "x" represents the mean contouring time, and the horizontal line represents the median. Image created by the authors with MS Excel (Microsoft Corp., WA, USA)

Streak artifacts adversely affected the auto-contouring performance of several structures, as shown in Figure 5. Nevertheless, the outlines of the influencing structures remained discernible, allowing manual modification of the contours on the CBCT. Contouring times, measured from acquisition of the first CBCT to completion of contouring, are shown in Figure 6 for four prostate bed patients over 25 fractions. Patient 1, who had a unilateral hip implant, was compared with three patients without implants (patients # 4-6), and no significant difference in contouring time was observed. In addition, the DSC and MHD for the OARS showed strong agreement, except for the bladder, which exhibited the highest mean MHD, as shown in Table 3.

**Table 3:** Comparison of the degree of agreement between OAR contours obtained by AI and those modified by physicians for patients 1 and 4-6 during the online on-couch adaptive session, using the mean dice similarity coefficient (DSC ± standard deviation) and mean maximum Hausdorff distance (MHD ± standard deviation) The DSC values range from 0 to 1, where higher values indicate better agreement. MHD quantifies the distance between structural boundaries; lower values indicate better agreement. OAR: organ at risk, AI: artificial intelligence, DSC: dice similarity coefficient, MHD: maximum Hausdorff distance

	Bladder	Bowel space	Rectum
DSC	0.91 ± 0.06	0.99 ± 0.00	0.96 ± 0.02
MHD (mm)	24.39 ± 11.45	7.56 ± 1.19	7.08 ± 4.33

## Discussion

oART is an emerging treatment paradigm that aims to restore optimal dose distributions based on the patient’s daily anatomy. CBCT-guided oART is an accessible platform that is rapidly gaining clinical traction. While the clinical implementation and utility of CBCT-guided oART have been widely reported, there are limited data describing its use in patients with MIs. This study presents our institutional workflow, focusing on dosimetric performance and additional considerations relevant to treating these patients with CBCT-guided oART.

Several studies have investigated radiation treatment planning in the presence of MIs. Su et al. compared IMRT with three-dimensional conformal radiation therapy (3D-CRT) incorporating prosthetic beam avoidance in patients with bilateral hip implants, demonstrating improved dose conformity and OAR sparing with IMRT [8]. Subsequent work showed that volumetric modulated arc therapy (VMAT) outperformed IMRT and 3D-CRT in prostate treatments, with superior conformity and greater rectal dose sparing [9]. Nadhum et al. explored four planning strategies for patients with hip implants: full VMAT arcs, arcs with avoidance sectors, arcs with avoidance sectors combined with lateral static fields passing through the implants, and full arcs with structure or implant avoidance [3]. Plans employing avoidance sectors alone demonstrated inferior target coverage and OAR sparing, whereas full arcs with structure avoidance were the most robust. As expected, unrestricted plans resulted in greater dose deposition through the prosthesis and higher doses at the implant-tissue interface.

This study reports our institutional experience managing oART patients with various MIs, including spine stabilization hardware, hip prostheses, and dental implants. For hip implant cases, treatment plans were optimized in the Ethos TPMS using the IOE V1.0. Dosimetric avoidance of the implant was applied to minimize beams passing through the prosthesis, in accordance with TG-63 recommendations. This approach also limits the dose exiting through the implant. The mean dose through the implant was comparable between scheduled and adaptive on-couch treatments, supporting the feasibility of dosimetric avoidance in an oART workflow. Although a statistically significant difference in mean Dmax was observed between scheduled and adaptive fractions, the absolute dose difference was less than 1 cGy per fraction. In this study, the dosimetric avoidance constraint was D0.03cc50% of the prescription dose. Stricter dose limits, as applied in the bilateral hip implant case, may further reduce implant dose but could redirect dose to anterior and posterior structures, potentially increasing rectal and bowel doses. As these cases represent the institution’s first experience with adaptive therapy in implant patients, two different avoidance strategies were explored to assess feasibility.

In our experience, overriding metal artifacts resulted in dose differences of approximately 1-2%, except in cases where the artifact directly overlapped the tissue of interest. Larger overlaps corresponded to greater dosimetric effects. Although dose measurements around the implants were not directly verified in this study, prior investigations have evaluated dose accuracy in and around MIs [2,4,8,10]. Pawalowski et al. [11] and Ojala et al. [12] assessed the accuracy of several dose calculation algorithms, including the analytical anisotropic algorithm (AAA), Acuros XB (AXB), and Monte Carlo (MC), in water phantoms with metal inserts. AAA was shown to inadequately model backscatter dose, whereas AXB, which employs a dose-to-medium formalism, demonstrated better agreement with MC. The Ethos IOE utilizes AXB as its dose calculation engine, enabling acceptable dose accuracy without explicit metal artifact override and thereby supporting a practical ART workflow for patients with MIs. In addition, the propagation of artifact contours across daily adaptive sessions closely matched those on the reference CT, indicating the feasibility of contouring and HU overriding for artifacts.

Streak artifacts in the absence of MAR can substantially degrade auto-contouring accuracy for influencer structures. Although iterative CBCT (iCBCT) reduces image noise and improves overall image quality [13], it remains insufficient for artifact removal. The presence of air in the abdomen or rectum can further exacerbate streak artifacts, leading to degraded image quality. Despite these limitations, both influencer and target structures could be manually modified on daily CBCT images. Notably, auto-contouring inaccuracies were observed even in cases without metal artifacts. The time required to edit contours did not differ significantly between patients with and without implants. DSC analysis demonstrated strong agreement between edited and non-edited AI-generated contours, with the exception of the bladder, which was more susceptible to artifact-related degradation.

This study is limited by the small number of patients included to demonstrate the proposed workflow. Therefore, the applicability and generalizability of the workflow may vary depending on the clinical site, the specific algorithm implemented, the imaging panel utilized, and institutional protocols. Careful evaluation and validation are warranted prior to broader clinical implementation. Additionally, the CT simulation images containing metal artifacts exhibited reduced image quality, as the simulator used in this study was not equipped with a metal artifact reduction (MAR) algorithm. Improved CT image quality in the presence of metal artifacts may influence workflow adaptation and potentially enhance overall performance.

The recent introduction of HyperSight on the Ethos platform represents a significant advancement in mitigating metal artifacts through improved image quality and an enhanced MAR algorithm [14]. The patients included in this study were treated prior to the availability of HyperSight; therefore, our findings provide practical guidance for clinics operating without this capability. With HyperSight, the ART workflow for these patients may become even more streamlined and robust, pending further investigation. Nonetheless, despite the presence of artifacts, the image quality of the earlier-generation Ethos CBCT was sufficient to visualize the outlines of influencer structures, consistent with previously published reports [15].

## Conclusions

Our clinical experience treating patients with MIs has led to the development of an efficient and reproducible workflow that can be readily adopted at other institutions. The proposed workflow suggests that artifact overriding may not be necessary unless the artifact directly overlaps a structure of interest, including the target or an organ at risk. Dose delivery through the implant can be minimized using dosimetric constraints applied to the implant structure. Although streak artifacts can affect auto-contouring performance, the time required for manual contour adjustment is comparable to that for patients without MIs.
